# Call for Systematic Population-Based Cervical Cancer Screening: Findings from Community-Based Screening Camps in Tamil Nadu, India

**DOI:** 10.31557/APJCP.2019.20.12.3703

**Published:** 2019

**Authors:** Elangovan Vidhubala, Hemant Deepak Shewade, Anandan K Niraimathi, Sethupathy Ramkumar, Gomathi Ramaswamy, G Nagalekshmi, B Sankar Mahadeva

**Affiliations:** 1 *Nellai Cancer Care Center, Tirunelveli, (An unit of Udhavum Ullangal), *; 7 *Department of General Surgery, Tirunelveli Medical College Hospital, Tirunelveli District, Tamil Nadu, *; 2 *Fenivi Research Solutions, Chennai, *; 4 *The Union South-East Asia Office,*; 6 *National Centre of Excellence and Advanced Research on Anemia Control (NCEAR -A), Centre for Community Medicine, All India Institute of Medical Sciences (AIIMS), New Delhi, *; 5 *Karuna Trust, Bengaluru, Karnataka, India, *; 3 *International Union Against Tuberculosis and Lung Disease (The Union), Paris, France. *

**Keywords:** Community, based screening, early diagnosis, camp approach, cancer cervix, VIA, Pap smear, SORT IT

## Abstract

**Background:**

In India, systematic cervical cancer screening under the national programme is yet to cover the entire population and therefore opportunistic or camp based approach is commonly practiced screening mode currently. This study presents the proportion of screen-positive women [positive visual inspection of the cervix with acetic acid (VIA) and/or Papanicolaou (Pap) smear results] and its associated factors from a rural community-based cervical cancer screening conducted in a service setting.

**Methods:**

In this cross-sectional study involving record review, data was drawn from free screening camps conducted by a non-governmental organization in two rural districts of Tamil Nadu, India between March 2015 and March 2017. The associations were assessed using adjusted prevalence ratio with 95% confidence interval.

**Results:**

A total of 5,207 women were screened from 307 camps. The mean age was 39.5 years (SD: 8.6). At least one symptom was observed among 2,245 women (43.1%). Of 5,207 women, 19.4% (n=1,009, 95% CI: 18.3%, 20.5%) were screen-positive. Screen positivity in women <30 years was 19%. Age 31-45 years, age at marriage 18-21 years, age at first child birth less than 18 years and unhealthy cervix (on examination) were significantly associated with screen positivity.

**Conclusion:**

Reduction in the minimum age of screening from 30 to 21 years considering their marital status and parity, and intensifying awareness campaigns to attract asymptomatic women would be advantageous in early detection and prevention of cervical cancer. Service-based organizations may adopt systematic population-based screening to increase the coverage instead of camp approach.

## Introduction

India accounts for one-quarter of the worldwide burden of cervical cancer (Ferlay et al., 2012; Institute for health metrics and evaluation , 2011) and 17% of all cancer deaths among women aged between 30 and 69 years. Cervical cancer is the second most common malignancy among women in India (Bruni et al., 2017). It is estimated that cervical cancer will occur in approximately 1 in 53 Indian women during their lifetime compared with 1 in 100 women in developed world (Institute for health metrics and evaluation, 2011). 

Decline in cervical cancer mortality is being noted in developed countries due to widespread screening. Coverage of cervical cancer screening in developing countries is 19%, compared to 63% in developed countries. Due to poor coverage of screening in India irrespective of economic status (rich-6%; poor-4%) (Gakidou et al., 2008), the 5-year relative survival rate for cancer is low (46%) compared to other countries (Sankaranarayanan et al., 2010). Nearly 70% of cervical cancer cases in India are being diagnosed at an advanced stage (stage III or IV) (Nandakumar et al., 1995). Therefore, it is recommended to expand the screening where sufficient infrastructure and health system access exists (Gakidou et al., 2008). 

National Programme for Prevention and Control of Cancer, Diabetes, Cardiovascular Disease and Stroke (NPCDCS) instituted in 2010 to control and prevent non-communicable diseases including cancer is implemented in a phased manner and is yet to reach the entire population (NPCDCS, 2017). 

Cervical cancer screening by testing for human papillomavirus (HPV), Papanicolaou (Pap) and visual inspection of the cervix with acetic acid (VIA) are found to be effective in detecting precancerous conditions (World Health Organization, 2013). Despite the proven strategies, a majority of the women in rural settings do not get an opportunity to undergo screening for cervical cancer. The negligible number of screening programmes conducted in camp modes have also poor uptake due to various psychosocial, structural, cultural and religious factors (Devarapalli et al., 2018). 

In India, in a cross sectional community based study, the proportion of VIA positive was 5.5% and Pap-premalignant lesion was 3.6% (Arun et al., 2018). Similarly, in a tertiary hospital, 2% of the women screened had abnormal cytology (Dhanasekaran et al., 2019). Positivity rates were associated with age, parity, education, religion, caste, visually apparent cervical inflammation, marital status and early age at first intercourse and first child birth (Arun et al., 2018; Vedantham et al., 2010; Ibrahim et al., 2012; Gravitt et al., 2010; Bosch et al.1992; Nguyen et al., 2002; Ngoma et al., 2010; Bhattacharyya et al., 2015; Makuza et al., 2005; Dhanasekaran et al., 2019). 

Adsul et al., (2017) identified 20 studies on community-based cervical cancer screening (randomized clinical trials-3, cross sectional studies-17) in India; most of them studied the accuracy of screening tests. Adsul indicated the paucity of published data based on community-based cervical cancer screening conducted with service intent. Therefore, we aimed to determine the proportion of women who screened positive in a community-based cervical cancer screening programme (using both VIA and Pap testing) in rural areas of south India and the factors associated with screen-positive.

## Materials and Methods

*Study Design*

This was a cross sectional study involving record review of data from a community-based screening programme using camp approach.

*Settings*

The study was conducted in two southern districts of Tamil Nadu, namely Tirunelveli and Tuticorin. Nellai Cancer Care Center (NCCC – based at the district headquarters of Tirunelveli) had formal collaborations with local non-governmental organizations (NGOs) and many of the screening camps were conducted in liaison with NGOs in their field practice. In addition, few villages were included based on specific requests. Multiple camps were conducted in few villages to enhance the coverage.

**Figure 1:**
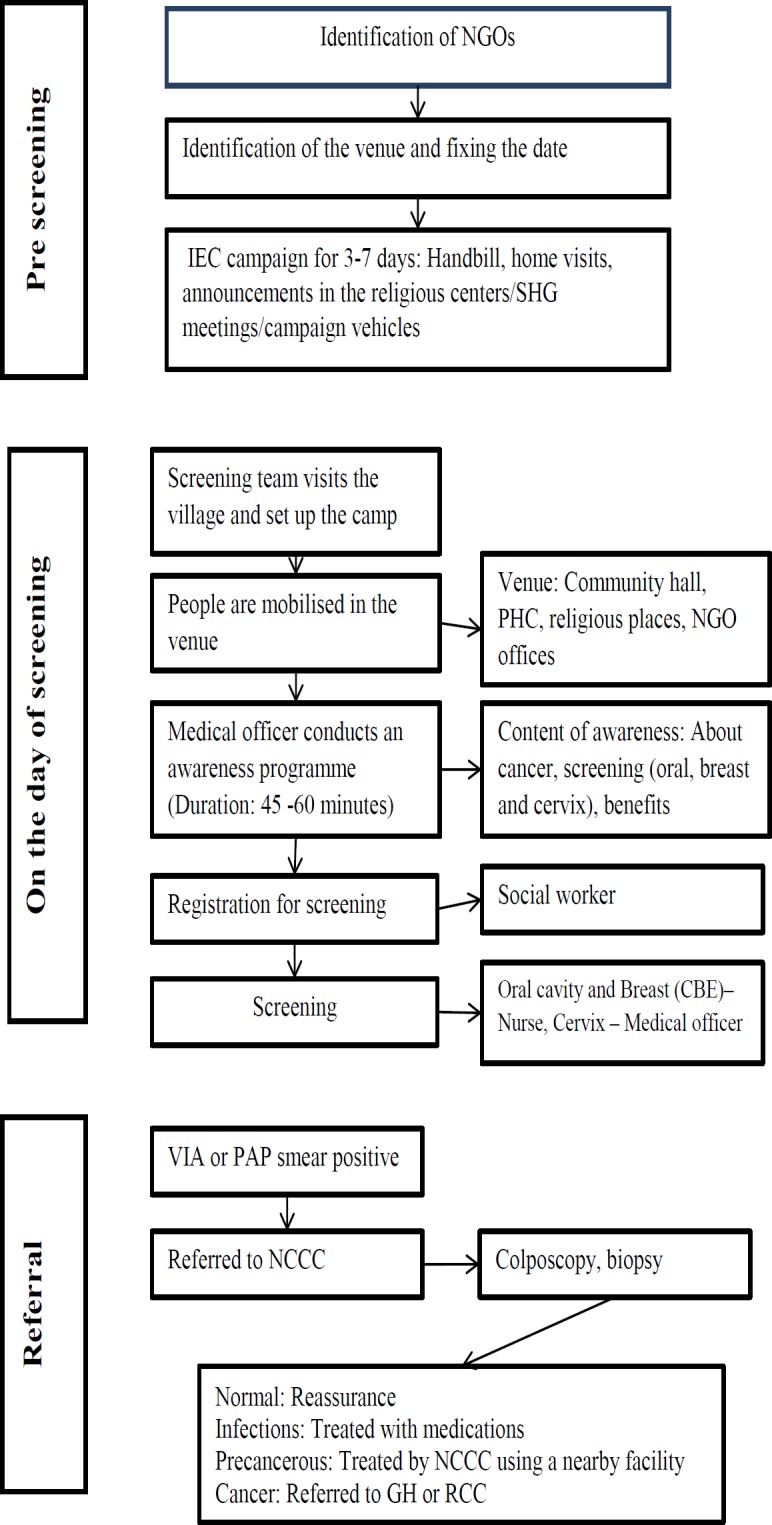
Process of Community-Based Screening Conducted by Nellai Cancer Care Center (Camp Approach), in Rural Areas of Tirunelveli and Tuticorin District, Tamil Nadu, India (March, 2015- March, 2017)

Note: NCCC- Nellai Cancer Care Center, CBE-Clinical Breast Examination, VIA- Visual Inspection after application of Acetic Acid, NGO-Non-Governmental organization, IEC- Information Education Communication, SHG-Self Help Group, GH-General Hospital, RCC-Regional Cancer Center.

The screening process is presented in Figure 1. The screening camps were led by a team of health professionals, including medical and paramedical staff, who were trained to conduct screening as per the WHO guidelines (WHO, 2013). Following the sensitization on cancer screening and prevention, villagers were encouraged to participate in the cancer screening program. Written and oral consent was obtained from the individuals who are willing to participate in the screening before registration. Breast, cervix and oral cavity were the sites screened. 

For cervical cancer screening, women who were healthy, currently or had been married, not pregnant, had an intact uterus with no prolapse, had no history of cervical cancer, and living in the camp areas were screened. At the time of registration, the demographic and clinical details were collected and documented as clinical case records. All the women were subjected to Pap smear followed by VIA. The VIA results were classified as negative and positive (International Agency for Research on Cancer, 2005). The fixed slides were transported to NCCC for Pap staining based on the conventional method. For those smears found to be satisfactory for evaluation, cytological abnormalities were recorded based on the Bethesda System (Annexure 1 and 2) (Nayar and Wilbur, 2015). The slides were examined and results were reported by the pathologist. Any women who found to have abnormality based on either Pap or VIA results were referred to NCCC for further evaluation. Additional criterion for referral was woman whose specimen was unsatisfactory or inadequately stained or broken for repeat cytology screening. 

Women with reported abnormalities underwent colposcopy examination and endocervical curettage, if required. Women with pre-malignant lesions [Cervical Intraepithelial Neoplasia (CIN)-I, II, III], were treated and followed up at NCCC. Women with malignancy were referred to regional cancer centres or the government hospitals. The women with infections were treated with antibiotics and advised for follow up after one year.

The community-based screening (VIA, Pap smear testing) and further investigations such as colposcopy, biopsy and medicines are provided for those patients with infections (at NCCC) free of cost. Full or partial financial support for treatment is provided by NCCC based on the socio-economic status of the patients. The clinical staff at NCCC liaison with the oncologists at regional cancer centres or the government hospitals and facilitate the investigation and treatment process until treatment completion and ensure further follow-up throughout survivorship. Despite these efforts, anecdotal evidence suggests that turn out rate for screening and follow-up is minimal. 

*Study population*

All women screened for cervical cancer between March 2015 and March 2017 under the community-based screening camps organised by NCCC in rural areas of Tirunelveli and few villages in Tuticorin district that lie adjacent to Tirunelveli district were included in the study. 

*Data variables, sources of data and data collection*

The demographic and clinical variables such as age, marital status, age at menarche, religion, education, occupation, age at marriage, parity, age at first child birth, tobacco use, signs and symptoms, cervix hygiene and VIA results were extracted from the clinical case records. The Pap results were extracted from the laboratory register at NCCC. Data were collected using a structured data collection form.

*Analysis and statistics*

Data were single-entered in EpiData version 3.1 (EpiData Association, Odense, Denmark). Data was analysed using Stata Version 15.0 (StataCorp, College Station, Texas, USA). The socio-demographic, parity related characteristics and clinical characteristics were summarized as number and proportion. The study participants with negative results in both VIA and Pap smear were categorized as normal/negative for cervical screening (Annexure 3). Those with positive for precancerous/cancerous conditions in any of the tests (VIA or Pap smear) were considered as positive for cervical screening. Though VIA is recommended for low resource settings, co-testing has proved to be clinically and economically beneficial (Qureshi et al., 2010; Blatt et al., 2015; Felix et al., 2016).

Modified Poisson regression (adjusted analysis) with robust variance estimation was used to assess the association of factors (socio-demographic characteristics, parity, clinical characteristics) with screen-positivity. Complete case analysis was done as data was missing at random for certain factors. Factors with p value <0.2 in unadjusted analysis were included in the adjusted analysis. Multicollinearity was ruled out before including the factors in the regression model. Prevalence ratios (unadjusted and adjusted – PR and aPR) with 95% confidence interval (CI) were calculated as measure of association. 

*Ethics*

Ethics approval was obtained from the Ethics Advisory Group of the International Union against Tuberculosis and Lung Disease, Paris, France (EAG Number-92/18, dated 02 November 2018). Permission to conduct the study using the clinical data was obtained from NCCC. As this study involved review of routinely collected secondary data, waiver for informed consent was sought and approved by the ethics committee.

## Results

**Figure 2:**
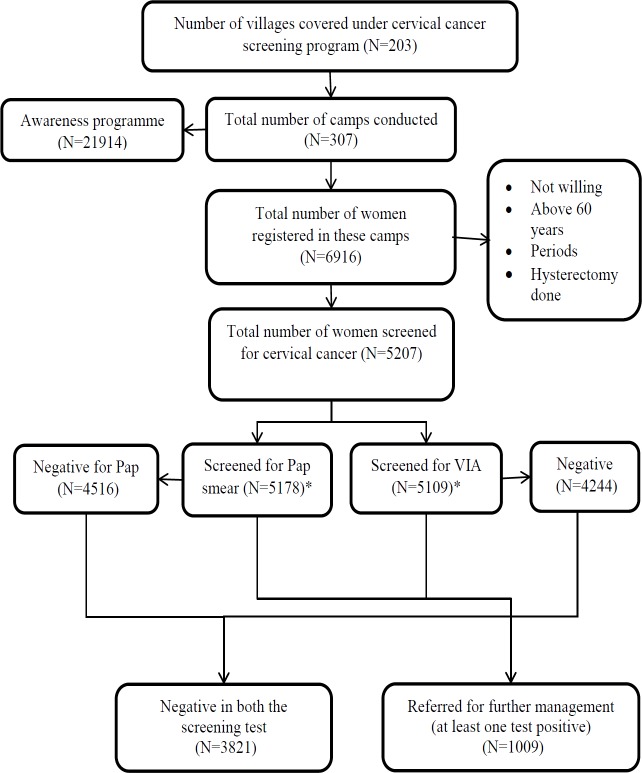
Flowchart Depicting the Proportion of Screen-Positive from the Community-Based Cervical Cancer Screening Camps Conducted by NCCC between March 2015 and March 2017 in Rural Areas of Tirunelveli and Tuticorin District, Tamil Nadu, India. Note: NCCC- Nellai Cancer Care Center, VIA- Visual Inspection after application of Acetic Acid; (* the number of women screened for Pap smear and VIA is not mutually exclusive, VIA results were not available for 98 women, Pap smear results were not available for 490, both positive=57)

The data was drawn from 307 screening camps conducted in 203 villages. Of the women sensitized for cancer screening at the camp site (n=21,914), 6916 (31.6%) registered for screening. The attendance per camp ranged from 2-48 (Mean-21.6, SD-9.1). Of them, 5,207 patients were screened for cervical cancer: mean 17 per camp (Figure 2). Socio-demographic characteristics of 5207 women undergoing screening are shown in Table 1. Majority were in age group of 30-45 years with mean age of 39.5 years (SD: 8.6). Of the women screened, 1,133 (21.8%) women were engaged in bidi rolling. Age at marriage and first child birth below 18 years was 23.2% and 9.1% respectively. More than two pregnancies were reported by 59% of women. 

Table 2 depicts the presenting signs and symptoms of the women who attended screening for cervical cancer. Of the women screened, 2,245 women (43.1%) had at least one symptom and the most common presenting symptom reported was white discharge per vaginum (n=2,170; 96.6%). Unhealthy cervix was documented in 1,783 (34.2%) and cervical erosion was reported in 1,533 (29.4%) women.

**Figure 3:**
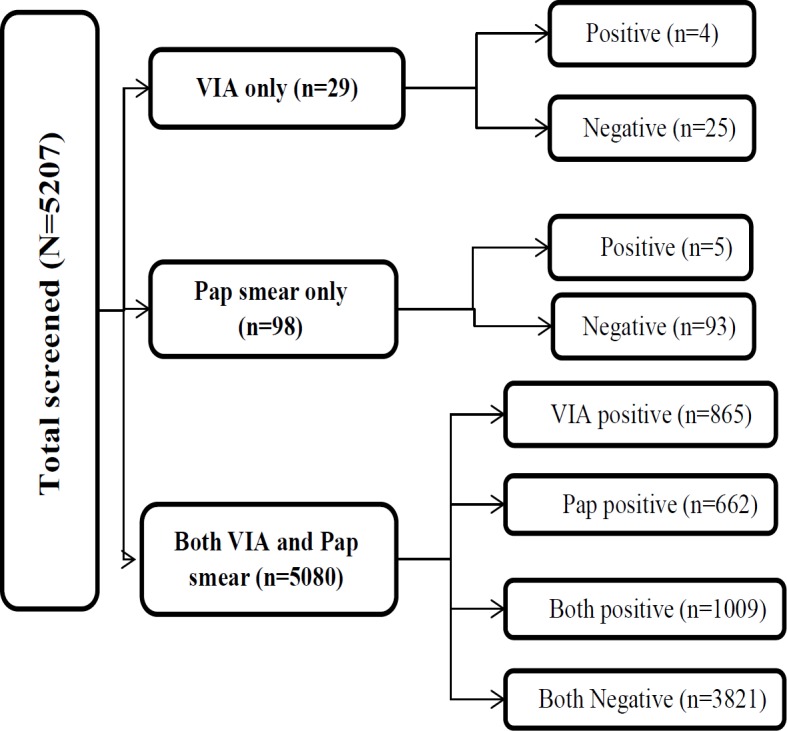
Flowchart Depicting the Results of Screening Tests from the Community-Based Cervical Cancer Screening Camps Conducted by NCCC between March 2015 and March 2017 in Rural Areas of Tirunelveli and Tuticorin District, Tamil Nadu, India. Note: NCCC- Nellai Cancer Care Center, VIA- Visual Inspection after application of Acetic Acid

Of 5,207 women, 19.4% (n=1009, 95% CI: 18.3%, 20.5%) were screen-positive (Figure 2). Screen positivity in women <30 years was 19%. A total of 865 (16.6%) women were VIA positive and 201 (3.9%) women were Pap positive (Table 2, Figure 3). Both the tests were positive among 57 (1%) women. Age 31-45 years, age at marriage 18-21 years, age at first child birth less than 18 years and unhealthy cervix (on examination) were significantly associated with screen positivity (Table 3). 

## Discussion

This study is based on the community-based cervical cancer screening using camp approach in service setting in rural areas of South India. One in two women presented with at least one symptom and one in five women was screen-positive. Certain categories of women who were at higher risk of screening positive were detected. 

The new UN Global Joint Programme is working towards tackling cervical cancer to contribute to reaching the 2030 Agenda for Sustainable Development Goals in terms of ensuring healthy lives and reducing inequalities within and among countries (US Preventive Services Task Force, 2018). It recommends the government of every country to strengthen and build research capacity to support decisions on country-adapted screening and treatment algorithms. This study presents newer evidence from the actual community-based screening conducted with the service motive. While the Indian Government is currently implementing the population-based cervical cancer screening programme under NPCDCS (NPCDCS, 2017), the findings from this study will be useful to prioritize the programme in high prevalence districts.

**Table 1:** Socio-Demographic and Clinical Characteristics of Women who Attended Cervical Cancer Screening Programme Conducted by NCCC between March 2015 and March 2017 in Rural Areas of Tirunelveli and Tuticorin District, Tamil Nadu, India

Characteristics	N	(%)
Total	5,207	(100)
Age in years		
<30	779	(15)
30(45	3,135	(60.2)
45(60	1,235	(23.7)
>60	53	(1.0)
Not recorded	5	(0.1)
Marital status		
Married	5,138	(98.7)
Never married*	12	(0.2)
Divorced /Separated /Widow	38	(0.7)
Not recorded	19	(0.4)
Education		
No formal education	953	(18.3)
Primary schooling	1,479	(28.4)
Secondary schooling	1,869	(35.9)
Higher secondary	360	(6.9)
Degree or above	519	(10)
Not recorded	27	(0.5)
Occupation		
Unemployed	1,811	(34.8)
Employed	2,258	(43.4)
Bidi workers	1,133	(21.8)
Not recorded	5	(0.1)
Religion		
Hindu	3,980	(76.4)
Muslims	293	(5.6)
Christians	917	(17.6)
Others	5	(0.1)
Not recorded	12	(0.2)
Age at menarche in years		
≤12	493	(9.5)
13 (15	3,561	(68.4)
>15	1,153	(22.1)
Age at marriage in years		
<18	1,210	(23.2)
18(21	2,110	(40.5)
>21	1,887	(36.2)
Parity		
Nulliparous	10	(0.2)
Parous	5,197	(99.8)
Age at first child birth in years		
<18	475	(9.1)
19-30	1,674	(32.1)
>30	3,004	(57.7)
Not recorded	54	(1.0)
Total pregnancies		
≤2	2,149	(41.3)
>2	3,058	(58.7)
Number of children		
≤2	3,211	(61.7)
>2	1,993	(38.3)
Not recorded	3	(0.1)

NCCC- Nellai Cancer Care Center, * These are the women aged above 50 years and considering their age as a risk factor they were included in the study even though they were not married.

**Table 2:** Clinical Symptoms, Signs, VIA and Pap Smear Results of Women who Attended Cervical Cancer Screening Camps Conducted by NCCC between March 2015 and March 2017 in Rural Areas of Tirunelveli and Tuticorin District, Tamil Nadu, India

Symptoms and Signs	N	%
Total	5,207	(100)
Symptoms		
Not recorded	154	(3.0)
No complaints	2,808	(53.9)
Complaints present*	2,245	(43.1)
White discharge per vaginum	2,170	(96.6)
Bleeding per vaginum	54	(2.4)
Urinary symptoms	92	(1.8)
Cervix		
Healthy cervix	3,152	(60.5)
Unhealthy cervix	1,783	(34.2)
Not recorded	272	(5.2)
Sign		
Cervicitis	86	(1.7)
Nabothian follicles	162	(3.1)
Cervical Erosion	1,533	(29.4)
Cervical Leukoplakia	33	(0.6)
Condyloma	8	(0.2)
Vaginal vault lesion	3	(0.1)
Growth invasive cancer	18	(0.3)
Cervix polyp	137	(2.6)
Bleeding on touch	144	(2.8)
VIA Results		
Positive	865	(16.6)
Negative	4,244	(81.5)
Not done/not recorded	98	(1.9)
Pap smear results		
Negative	4,516	(86.7)
Precancerous	198	(3.8)
Cancerous	3	(0.1)
Unsatisfactory/inadequate staining/broken	461	(8.9)
Not recorded	29	(0.6)

NCCC, Nellai Cancer Care Center; VIA, Visual Inspection of Acetic Acid; *Denominator, Presence of symptoms

**Table 3:** Factors Associated with Screen Positivity from Community-Based Cervical Cancer Screening Camps Conducted by NCCC between March 2015 and March 2017 in Rural Areas of Tirunelveli and Tuticorin District, Tamil Nadu, India

Parameters	Pap and VIA results			Unadjusted PR (95%CI)	Adjusted PR (95%CI)
	Total	Screen	Positive Ϯ Ϯ		
	N	N	%#		
Total	4500Ϯ	945 Ϯ	(21.0)		
Age in years					
≤30	667	127	(19.0)	Reference	Reference
31-45	2,720	624	(22.9)	1.20 (1.0-1.42)	1.27 (1.07-1.51)*
46-60	1,079	189	(17.5)	0.91 (0.75-1.12)	1.02 (0.82-1.26)
>60	34	5	(14.7)	0.77 (0.33-1.76)	0.83 (0.37-1.90)
Education					
No formal education	849	186	(21.9)	1.13 (0.90-1.43)	
Primary schooling	1,284	259	(20.1)	1.04 (0.84-1.30)	-^
Secondary schooling	1,625	362	(22.2)	1.15 (0.93-1.43)	
Higher secondary	305	54	(17.7)	0.92 (.67-1.25)	
Degree or above	437	84	(19.2)	Reference	
Religion					
Hindu	3481	713	(20.5)	Reference	Reference
Muslims	240	65	(27.0)	1.32 (1.06-1.64)	1.21 (0.98-1.50)
Christians	779	167	(21.4)	1.04 (0 .90-1.21)	1.08 (0.93-1.26)
Employment status					
Unemployed	1,570	365	(23.2)	1.07 (0.92-1.24)	1.09 (0.92-1.23)
Employed	1,909	359	(18.8)	0.87 (0.75-1.01)	0.87 (0.75-1.01)
Bidi workers**	1,021	221	(21.6)	Reference	Reference
Age at menarche in years					
≤12	411	90	(21.9)	1.04 (0.85-1.26)	-^
13 -15	3,083	647	(20.9)	Reference	
>15	1,006	208	(20.6)	0.98 (0.85-1.13)	
Age at marriage in years					
<18	1,057	249	(23.5)	1.36 (1.16-1.58)	1.05 (0.84-1.30)
18-21	1,842	419	(22.7)	1.31 (1.14-1.50)	1.20 (1.02-1.41)*
>21	1,601	277	(17.3)	Reference	Reference
Age at first childbirth in years					
<18	415	111	(26.7)	1.43 (1.20-1.71)	1.41 (1.11-1.80)*
18-21	1,471	344	(23.3)	1.25 (1.11-1.42)	1.13 (0.97-1.31)
>21	2,584	481	(18.6)	Reference	Reference
Number of children					
≤2	2,747	546	(19.8)	Reference	Reference
>2	1,750	398	(22.7)	1.14 (1.02-1.28)	1.10 (0.98-1.24)
Symptoms					
Present	2,008	476	(23.7)	1.25 (1.12-1.41)	1.05 (0.93-1.18)
Not present	2,492	469	(18.8)	Reference	Reference
Healthy cervix					
Healthy	2,931	455	(15.5)	Reference	Reference
Not healthy	1,569	490	(31.2)	2.01 (1.79-2.24)	1.92 (1.71-2.15)*

NCCC, Nellai Cancer Care Center; VIA, Visual Inspection after application of Acetic Acid; PR, Prevalence Ratio; Ϯ Ϯ, either VIA or Pap smear positive; Ϯ, Missing data was excluded using complete case analysis; ^, not included in model as unadjusted p < 0.2; #, Row percentage; *, Statistically significant; **, Home based workers

High prevalence of screen-positive suggests a significant burden of potential cervical cancer cases. The prevalence of screen positivity was one in five, which is higher compared to other studies from low income countries including India (Vedantham et al., 2010). In our study, the response to camp-based approach was poor and half the women were those with symptoms. Therefore to improve the coverage and screening of asymptomatic women, population-based systematic screening is recommended. 

It is recommended that cervical cancer screening should begin at 21 years of age, with cervical cytology testing exclusively until age 30 years (US Preventive Services Task Force, 2018). In our study, 15% were <30 years of age and among them 19% were screen-positive. Women of all age group, keeping marital status as the criteria were included in the screening considering the early marital status and early child birth in our population. Keeping these results in mind and SDG’s main agenda of ‘no one to be left behind’, NPCDCS recommendations (currently screening is recommended for >30 years) may be revised to include women above 21 years of age especially if they are married and/or sexually active. However, this will incur greater allocation of resources in terms of infrastructure, training, ‘information education and communication’ activity and incentives.

Among the screened women, more than one-fifth women were bidi-workers. They have long working hours, continuous sitting work posture, exposure to tobacco dust, poor physical working conditions and non-adherence to personal protection measures (Reddy and Gupta, 2004; Sabale et al., 2012; Shukla et al., 2011; Yasmin et al., 2010; Nag et al., 2016). Evidences are emerging linking bidi-work and cervical cancer. In this study, non-association between bidi-work and screen positivity was due to misclassification bias. Many, who reported not currently involved in bidi-work, could be past bidi-workers and many who reported to be currently involved in bidi-work, could have started bidi-work only recently. This could nullify any association that could be present in reality. Also, duration of bidi-work could have helped in assessing the dose-response relationship. Future studies should collect information on past as well as current bidi-work status along with duration. 

Tobacco and alcohol usage was associated with VIA/Pap positivity in the western literatures. In this study population, the use of smokeless tobacco (1.3%) and alcohol (one woman) was found to be very low. 

There were some limitations in our study. Baseline data included variables collected at the time of screening. We did not collect detailed information related to bidi work. Data on nutritional status (weight and haemoglobin) was not available. Temporality in the association between patient characteristics and screen-positivity cannot be ascertained and this is a known limitation of cross-sectional design. The outcome of screen-positives is limited by the fact that only 10% of the screen-positive attended NCCC for further investigation and confirmation (data not shown). 

In this community-based cervical cancer screening (using VIA and Pap smear) conducted in South India using a ‘camp approach’, the proportion of women who screened positive was high compared to other settings. A large proportion of women less than 30 years screened positive and this has implication for the NPCDCS to reduce the age cut off from 30 years to the international recommendation of 21 years. There is a need for strategies to encourage asymptomatic women to participate in screening and improve coverage through population-based systematic screening (instead of camp approach); however this has to be matched with enhancing infrastructure and facilities for managing the screen-positive patients. Future studies from this area should include detailed information on bidi-work to explore the association between bidi-work and screen positivity. 

*Role of Investigators*

Conception / design of the protocol: EV, HDS, NA, ARD, SM; Acquisition of data, data analysis / interpretation: EV, GR, NA, HDS; Drafting /critically reviewing the paper, giving approval for the final version to be published: EV, HDS, NA, ARD, GR, SM, RS, GN; Role of SORT IT mentor: HDS, ARD, GR; Senior author: SM 
